# Functional matrix engineering enables aggregate-state photophysics in tumor-responsive organic NIR-II phototheranostics

**DOI:** 10.1016/j.mtbio.2026.103575

**Published:** 2026-08-19

**Authors:** Lei An, Changjin Ou, Honghuaijin An, Hanjun Sun, Xiaozhou Mou, Xiaochen Dong, Yu Cai

**Affiliations:** a Center for Rehabilitation Medicine, Rehabilitation & Sports Medicine Research Institute of Zhejiang Province, Department of Rehabilitation Medicine, Cancer Center, Zhejiang Provincial People's Hospital (Affiliated People's Hospital), Hangzhou Medical College, Hangzhou, Zhejiang, 310014, China; b Institute of Advanced Materials and Flexible Electronics (IAMFE), School of Chemistry and Materials Science, Nanjing University of Information Science and Technology, Nanjing, China; c State Key Laboratory of Flexible Electronics (LoFE), Institute of Advanced Materials (IAM), School of Flexible Electronics (Future Technologies), Nanjing Tech University (NanjingTech), Nanjing, 211816, China

**Keywords:** NIR-II phototheranostics, Functional matrix engineering, Assembly engineering, Intermolecular organization, Aggregate-state photophysics

## Abstract

Organic NIR-II phototheranostic nanoparticles function as molecular assemblies, making their optical performance fundamentally dependent on aggregate-state photophysics governed by intermolecular organization. However, exploiting the matrix to actively program intermolecular organization during nanoparticle assembly remains largely unexplored. Here, we establish functional matrix engineering as a practical implementation of assembly engineering, in which tumor-microenvironment-responsive prodrugs serve as multifunctional matrix components that reorganize intermolecular packing to enhance aggregate-state photophysics while simultaneously enabling therapeutic activation. A benzobisthiadiazole (BBT)-based D–A–A–D fluorophore (2A895) was co-assembled with two tumor-microenvironment-responsive prodrugs (CPB and Dyl) serving as multifunctional matrix components. Molecular dynamics simulations reveal that the functional matrix reorganizes intermolecular packing by disrupting compact π–π contacts, and indicates reduced electronic coupling through enlarged centroid separation and non-parallel packing, thereby suppressing aggregation-caused quenching and enhancing NIR-II fluorescence without compromising photothermal conversion. Meanwhile, the stimulus-responsive matrix enables H_2_O_2_-triggered chlorambucil release and concomitant GSH depletion, amplifying oxidative stress to potentiate chemo-photothermal therapy. The resulting nanoparticles exhibit enhanced NIR-II fluorescence and photoacoustic imaging together with robust in vivo antitumor efficacy. Overall, functional matrix engineering provides a general strategy for regulating molecular assemblies through matrix design rather than fluorophore redesign.

## Graphical abstract

**Figure:**
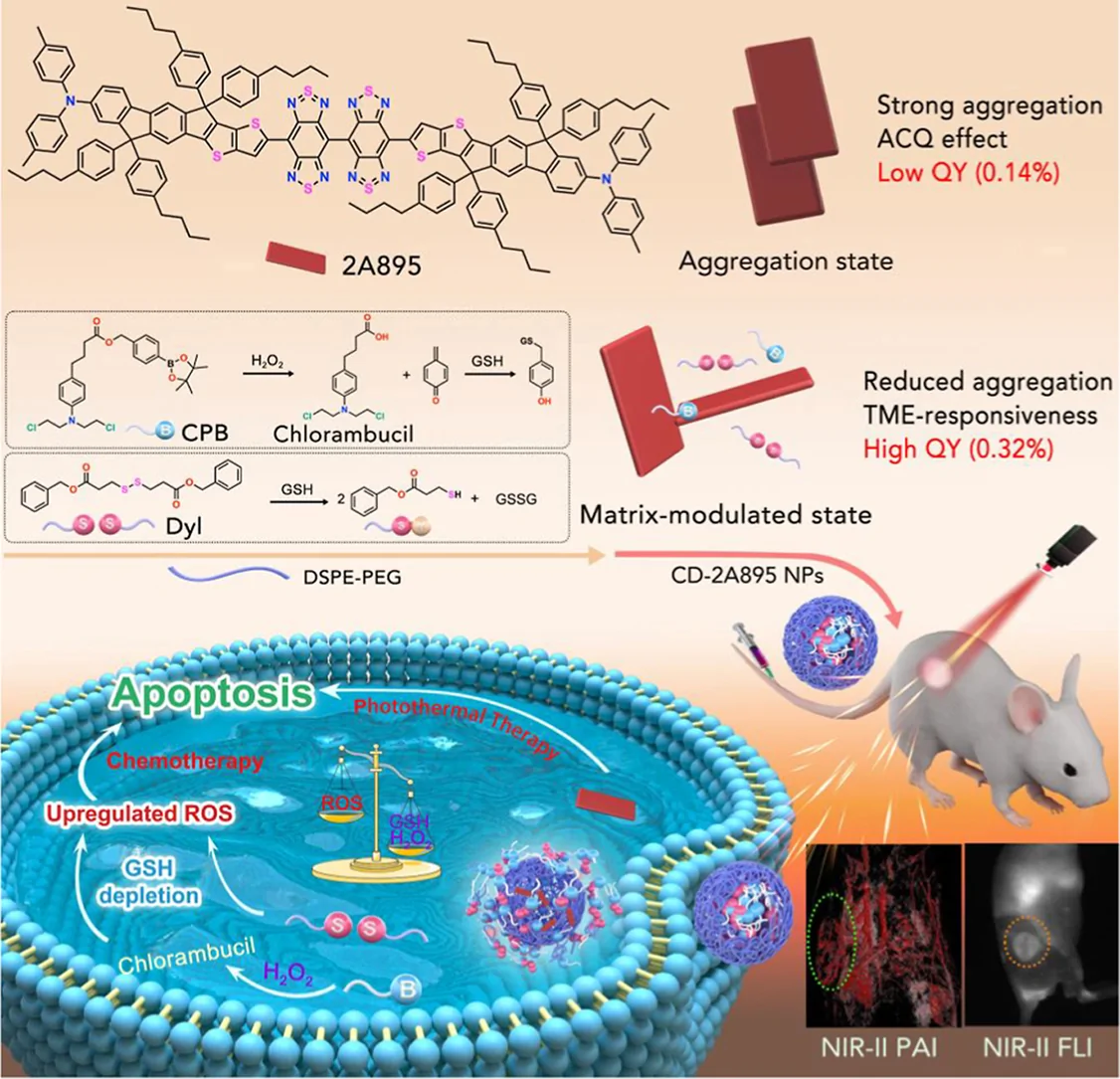

## 1 Introduction

The second near-infrared window (NIR-II, 1000–1700 nm) has attracted considerable interest for biomedical imaging because light in this spectral region experiences reduced photon scattering, weak tissue autofluorescence, and improved penetration depth [[1], [2], [3]]. Although organic NIR-II phototheranostic nanoparticles have advanced rapidly, optimizing their optical performance remains challenging because fluorophores inevitably assemble into molecular aggregates during nanoparticle formation, where their photophysical properties differ substantially from those of isolated molecules [[4], [5], [6], [7], [8], [9]]. The performance of the resulting nanoparticles is therefore determined not only by the intrinsic properties of the fluorophore itself, but also by the way molecules pack and interact within the assembled state. Understanding and controlling these intermolecular interactions has thus become a central issue in the development of high-performance NIR-II phototheranostic systems [[10], [11], [12], [13], [14], [15], [16]].

Within molecular assemblies, strong intermolecular π–π interactions establish extensive electronic coupling, promoting intermolecular exciton migration, intermolecular energy redistribution, and nonradiative decay that collectively lead to aggregation-caused quenching (ACQ) and diminished imaging performance [[17], [18], [19], [20], [21], [22]]. To alleviate these problems, extensive molecular engineering efforts have focused on fluorophore design, including conformational twisting, steric shielding, J-aggregate regulation, and donor–acceptor (D–A) optimization [[23], [24], [25], [26]]. These approaches effectively improve the intrinsic photophysical properties of individual molecules and reduce their tendency toward excessive aggregation. Nevertheless, molecular design alone cannot fully dictate how fluorophores ultimately organize after nanoparticle assembly. The final packing arrangement emerges collectively during the assembly process and is therefore only partially encoded by molecular structure.

Rather than redesigning fluorophores, assembly engineering offers a complementary strategy by manipulating intermolecular organization after nanoparticle formation. Among various approaches, matrix-assisted assembly has proven particularly effective because the surrounding matrix can reshape molecular packing and thereby influence aggregate-state photophysics [19,20,27]. Yet most reported matrix materials remain largely passive. They primarily function as carriers or physical spacers that dilute fluorophore packing and weaken excessive intermolecular interactions, while contributing little to the active regulation of excited-state processes. Their role is therefore largely confined to improving fluorescence performance, while contributing little to additional molecular functionality. Consequently, current assembly engineering has not yet evolved into a general strategy for simultaneously regulating aggregate-state photophysics and therapeutic function within molecular assemblies [18,25,28,29].

An important opportunity is therefore to extend assembly engineering beyond structural regulation toward multifunctional molecular programming, in which matrix components simultaneously regulate aggregate-state photophysics and therapeutic activation. This requires matrix components that actively participate in both molecular organization and biological function. Prodrug molecules are particularly attractive candidates because they combine structural compatibility for nanoparticle assembly with stimulus-responsive therapeutic functions [[28], [29], [30], [31]]. Meanwhile, the elevated glutathione (GSH) and hydrogen peroxide (H_2_O_2_) levels in the tumor microenvironment provide complementary opportunities for selective therapeutic activation [[32], [33], [34], [35], [36], [37], [38], [39], [40], [41], [42], [43]]. Nevertheless, integrating tumor-responsive therapeutic activation into assembly-engineered molecular assemblies while simultaneously regulating aggregate-state photophysics remains largely unexplored.

Herein, we report a functional assembly engineering strategy for constructing multifunctional organic NIR-II phototheranostic nanoparticles. A benzobisthiadiazole (BBT)-based D–A–A–D fluorophore (2A895) was developed as the NIR-II imaging and photothermal core, while two TME-responsive prodrugs (CPB and Dyl) were employed as functional matrix components. Unlike conventional inert matrices, the prodrug matrix reprograms intermolecular organization to modulate the aggregate-state photophysics, and enables GSH/H_2_O_2_-responsive therapeutic activation. Molecular dynamics simulations demonstrate that the functional matrix reorganizes intermolecular organization by disrupting compact π–π interactions, indicating reduced electronic coupling through enlarged centroid separation and non-parallel packing, thereby suppressing aggregation-caused quenching, and enhancing NIR-II fluorescence without sacrificing photothermal performance. The resulting nanoparticles further enable multimodal NIR-II fluorescence/photoacoustic imaging and effective chemo-photothermal therapy. More broadly, this work establishes a general design principle in which functional matrix engineering programs intermolecular organization to regulate aggregate-state photophysics and therapeutic function simultaneously, providing a versatile framework for engineering next-generation organic phototheranostic nanoparticles.

## 2 Results and discussion

### 2.1 Molecular construction and optoelectronic features

**Scheme 1:**
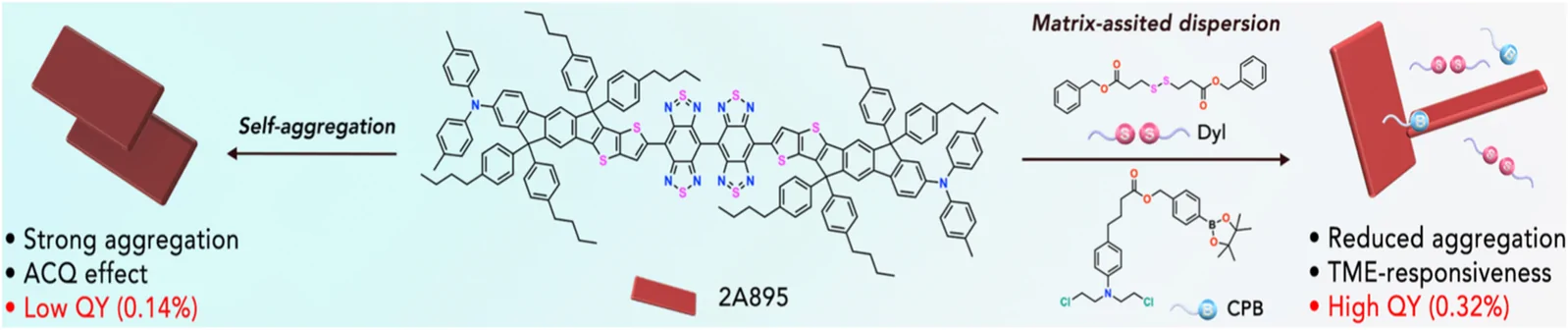
Design concept and therapeutic mechanism of the TME-responsive nanoparticles.

Benzobisthiadiazole (BBT), owing to its highly electron-deficient character, serves as an effective acceptor motif for the construction of long-wavelength NIR-II fluorophores [44,45]. Dual-BBT-based D-A-A-D molecules can adopt a planar–twisted–planar conformation that extends conjugation while mitigation excessive electronic coupling between neighboring chromophores. Guided by this molecular design concept, 2A895 was constructed by combining planarized donor units with a dual-BBT acceptor framework (Scheme 1). Scheme S1 illustrates the synthesis of 2A895. The chemical structures of all intermediates and the target fluorophore were confirmed by comprehensive NMR and MALDI-TOF analyses (Fig. S1-S9).

**Fig. 1:**
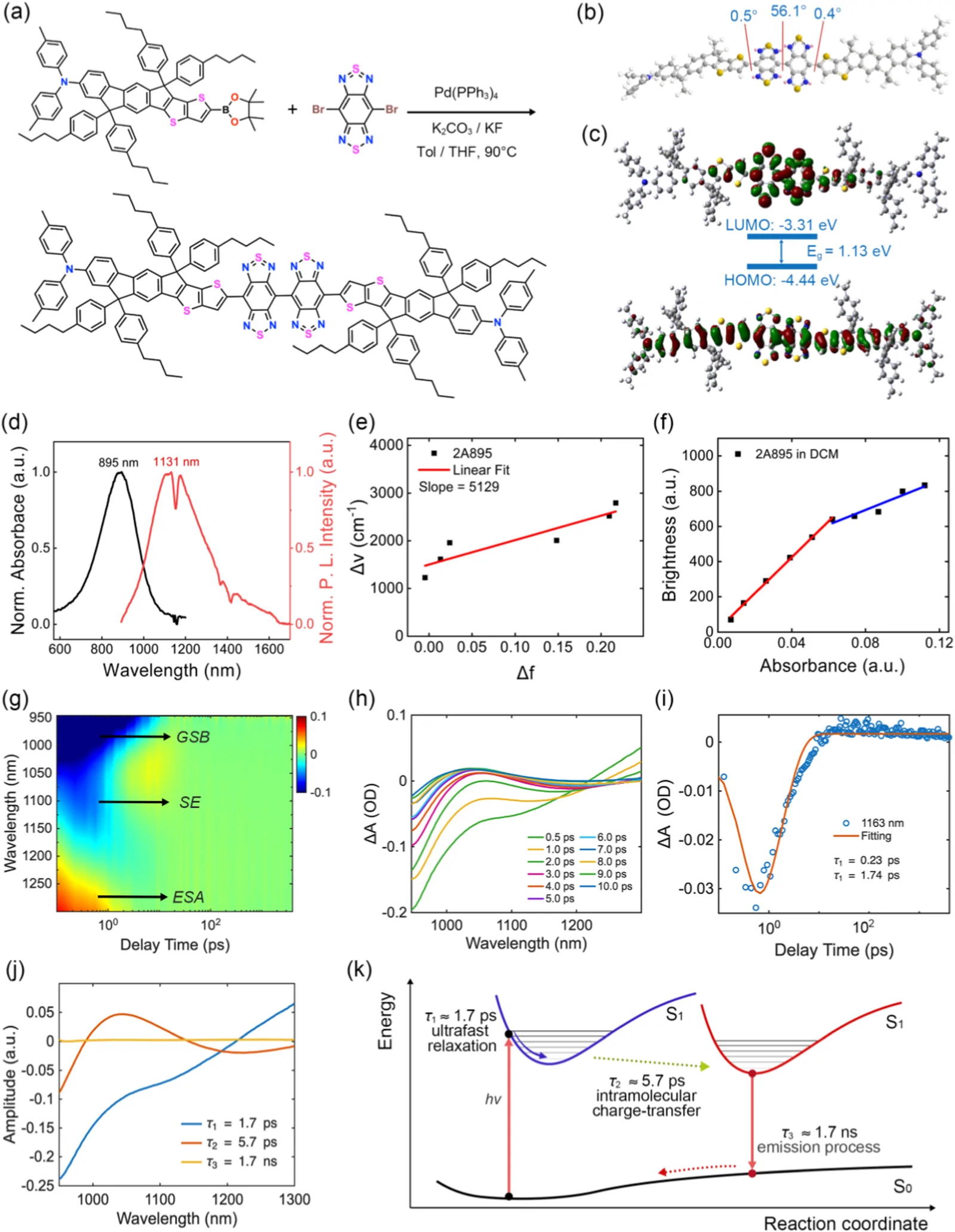
(a) Synthetic route toward the NIR-II fluorophore 2A895 through Suzuki-Miyaura coupling. (b) Energy-minimized molecular structure of 2A895 obtained using the B3LYP/6-31G(d) method. (c) Frontier molecular orbital distributions of 2A895 derived from DFT calculations. (d) Normalized optical spectra of 2A895, including absorption and emission profiles. (e) Lippert-Mataga analysis correlating the Stokes shift (Δν) with solvent polarity function (Δf). (f) Fluorescence brightness of 2A895 in DCM as a function of absorbance. (g) Femtosecond transient absorption contour map of 2A895 in DCM. (h) Transient absorption spectra at selected delay times. (i) Multi-exponential fitting analysis of characteristic wavelength at 1163 nm in stimulated emission area. (j) Species-associated spectra obtained from sequential global analysis with three kinetic components. (k) Proposed excited-state relaxation pathway mainly attributed to ultrafast relaxation, intramolecular charge-transfer and emission process.

Geometry optimization revealed that 2A895 adopts a characteristic planar–twisted–planar architecture, with torsion angles of 0.5°, 56.1°, and 0.4° across the conjugated backbone, respectively. (Fig. 1a and b). Frontier molecular orbital analysis reveals pronounced donor–acceptor electronic segregation, wherein the HOMO is delocalized over the conjugated backbone, while the LUMO is primarily confined to the dual-BBT acceptor moiety [46]. Such an orbital distribution promotes intramolecular charge transfer (ICT) and leads to a narrow electronic bandgap, with calculated HOMO/LUMO energy levels of −4.44 and −3.31 eV, respectively, corresponding to an energy gap of 1.13 eV (Fig. 1c). The ICT character of 2A895 is further supported by the positive linear correlation between the Stokes shift and solvent polarity (Fig. 1e, S10, Table S1). Consequently, 2A895 exhibits prominent NIR optical features, with an absorption maximum at 895 nm and fluorescence emission centered at 1131 nm in dichloromethane. (Fig. 1d). Strong light-harvesting capability is evidenced by high molar extinction coefficients of 4.25 × 10^4^ L mol^−1^ cm^−1^ and 2.34 × 10^4^ L mol^−1^ cm^−1^ at 895 nm and 980 nm, respectively. The fluorescence intensity increases linearly with absorbance at low concentrations but gradually deviates from linearity as the concentration increases (Fig. 1f, S11), indicating concentration-dependent fluorescence quenching. As the intermolecular distance decreases, interactions between neighboring fluorophores become increasingly pronounced, promoting intermolecular electronic coupling and intermolecular energy migration. The concentration-dependent evolution of fluorescence therefore illustrates how sensitive the photophysical properties of 2A895 are to its intermolecular packing, providing a strong rationale for regulating molecular organization during nanoparticle assembly.

The excited-state dynamics of 2A895 in dichloromethane are well described by a low-dimensional sequential relaxation model. The femtosecond transient absorption spectroscopy (fs-TAS) contour map displays pronounced positive and negative signals across 950–1300 nm, mainly concentrated in the early-time region (Fig. 1g and h). A representative kinetic trace at 1163 nm within the stimulated emission region was fitted by a two-exponential function yielding characteristic lifetimes of 0.23 ps and 1.74 ps. Global analysis using a three-state sequential kinetic model identified three consecutive time constants, namely 1.7 ps, 5.7 ps, and 1.7 ns, associated with the evolution of the excited-state processes (Fig. 1i and j). The population evolution further supports a sequential process, with rapid decay of component 1, rise-and-decay behavior of component 2, and gradual accumulation of a weak long-lived component 3 (Fig. S12, S13). As shown in Fig. 1k, these time constants are mainly attributed to the following physical processes: (i) intramolecular vibrational relaxation and solvation dynamics of ultrafast relaxation, (ii) intramolecular charge-transfer evolution and (iii) emission process [47]. Overall, the excited-state dynamics of 2A895 are consistent with efficient intramolecular charge transfer, ultimately leading to NIR-II emission.

### 2.2 Intermolecular organization of functional matrix assemblies

**Fig. 2:**
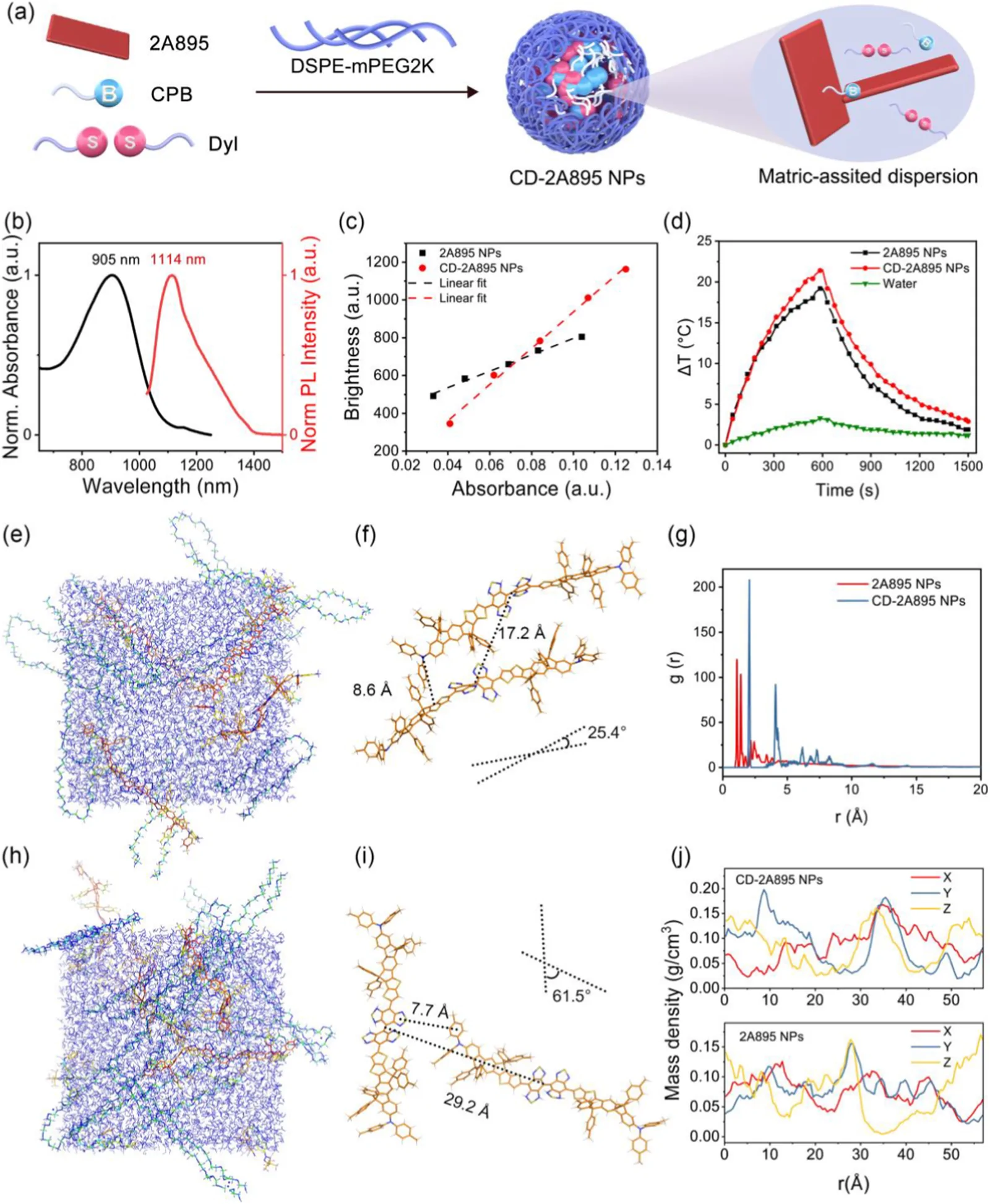
(a) Illustration of nanoparticle fabrication. (b) Absorption and emission characteristics of CD-2A895 NPs after spectral normalization. (c) Correlation between NIR-II signal output and absorbance for 2A895 NPs and CD-2A895 NPs, together with linear fitting results. (d) Temperature profiles of 2A895 NPs, CD-2A895 NPs, and water during laser irradiation and subsequent cooling (808 nm, 1.0 W/cm^2^). (e, h) Representative equilibrium configurations of 2A895 NPs and CD-2A895 NPs, respectively. (f, i) Enlarged views of local intermolecular arrangements extracted from the MD plot. (g) Radial distribution functions of 2A895 NPs and CD-2A895 NPs, respectively. (j) Mass density distributions along the X, Y, and Z directions for 2A895 NPs and CD-2A895 NPs, respectively.

To simultaneously regulate intermolecular π–π interactions and introduce chemotherapeutic functionality, two prodrug molecules, CPB and Dyl, were further designed as modular matrix components for nanoparticle assembly (Scheme S2, Fig. S14-S19). CPB, Dyl and 2A895 were encapsulated into water-soluble nanoparticles (named CD-2A895 NPs) by amphiphilic polymer DSPE-PEG2000. From single molecule to aggregate, the relative quantum yield (QY) decreased from 0.36% in dichloromethane to 0.14% in 2A895 NPs. The retention of fluoresnce is consistent with the planar-twisted-planar conformation and matrix modulation of intermolecular interactions alleviating ACQ effect (Fig. S20-S31, Table 1, Table S2) [20,28]. Impressively, despite exhibiting NIR absorption comparable to that of 2A895 NPs, CD-2A895 NPs showed a substantial increase in fluorescence efficiency, with the QY rising from 0.14% to 0.32%. Consequently, the fluorescence brightness (defined as ε×QY) more than doubled, increasing from 24.6 to 57.6 L mol^−1^ cm^−1^ under 980 nm excitation (Fig. 2a–c, Table 1). In addition, CD-2A895 NPs exhibited concentration-dependent photoacoustic responses and retained a photothermal conversion efficiency comparable to that of 2A895 NPs (29.8% versus 31.5%) (Fig. 2d, S32-S34, Table S3). Given the relatively loose molecular packing, the limited conformational change of 2A895 within the nanoparticles, and the narrow bandgap of 2A895 that keeps radiative efficiency low, excited-state energy remains dominated by intramolecular nonradiative decay. Therefore, modulation of intermolecular packing by the matrix is unlikely to significantly affect nonradiative decay channels. Instead, the observed fluorescence enhancement is attributed to alleviation of aggregation-associated quenching.

**Table 1:** Photophysical parameters of 2A895 and its NPs.

Sample	ε (10^4^ L·mol^−1^cm^−1^)^a^			QY^b^ (%)	ε×QY^c^ (L·mol^−1^cm^−1^)
	λ_808_	λ_Max_	λ_980_		
2A895	2.91	4.25	2.34	0.36	153.0/84.1
2A895 NPs	2.08	2.81	1.76	0.14	39.3/24.6
CD-2A895 NPs	2.10	3.01	1.80	0.32	96.3/57.6

a Molar extinction coefficient.

b Relative photoluminescence quantum yield.

c Fluorescence brightness, calculated as ε×QY using the molar extinction coefficients at the absorption maximum and at 980 nm, respectively.

To elucidate the mechanism underlying the enhanced fluorescence performance, molecular dynamics (MD) simulations were performed for both 2A895 NPs and CD-2A895 NPs (Fig. 2e and h) [48]. In 2A895 NPs, neighboring dye molecules tend to adopt relatively aligned packing configuration with a projected intermolecular angle of ∼25.4°. Although the shortest distance between neighboring conjugated segments is 8.6 Å, the centroid-to-centroid separation is relatively small (17.2 Å), indicating weak spatial overlap between the π-conjugated fluorophores (Fig. 2f). In contrast, after incorporation into CPB/Dyl matrix, the local packing arrangement of 2A895 is significantly altered in CD-2A895 NPs. The neighboring dye molecules adopt a non-parallel configuration with a much larger projected intermolecular angle of ∼61.5°. Meanwhile, although the shortest distance between conjugated segments becomes slightly smaller (7.7 Å), the centroid distance increases markedly to 29.2 Å (Fig. 2i). Trajectory-averaged analysis confirmed that the functional matrix changed the neighboring 2A895 molecules from a nearly parallel arrangement to a strongly nonparallel orientation, while only moderately increasing their centroid-to-centroid separation (Fig. S35). This structural modulation is further supported by the radial distribution functions (RDF) and mass-density analyses (Fig. 2g and j). As shown by the RDF profiles, the first correlation peak of CD-2A895 NPs shifts to a larger intermolecular distance compared with that of 2A895 NPs, indicating that the CPB/Dyl matrix effectively separates neighboring dye molecules and reduces direct dye–dye contacts. Meanwhile, the mass-density distributions reveal a reorganized internal packing environment in CD-2A895 NPs, suggesting that the matrix molecules are uniformly incorporated into the nanoparticle interior rather than forming segregated domains. Together with the enlarged centroid-to-centroid separation and non-parallel packing geometry, these results demonstrate that matrix-assisted assembly suppresses effective π-π overlap and intermolecular electronic coupling, thereby suppressing ACQ in the aggregate state [48,49]. So CD-2A895 NPs have a nearly identical relative QY with that of in dichloromethane. In addition, CPB and Dyl mainly form dimers through relatively weak van der Waals interactions (Fig. S36), which is beneficial for improving molecular miscibility with 2A895 via the “like dissolves like” rule and suppressing phase separation within the nanoparticles. These results suggest reduced effective intermolecular electronic coupling through enlarged centroid separation and non-parallel packing, thereby helping suppress ACQ effect in the aggregate state.

### 2.3 Tumor microenvironment-responsive therapeutic activation

**Fig. 3:**
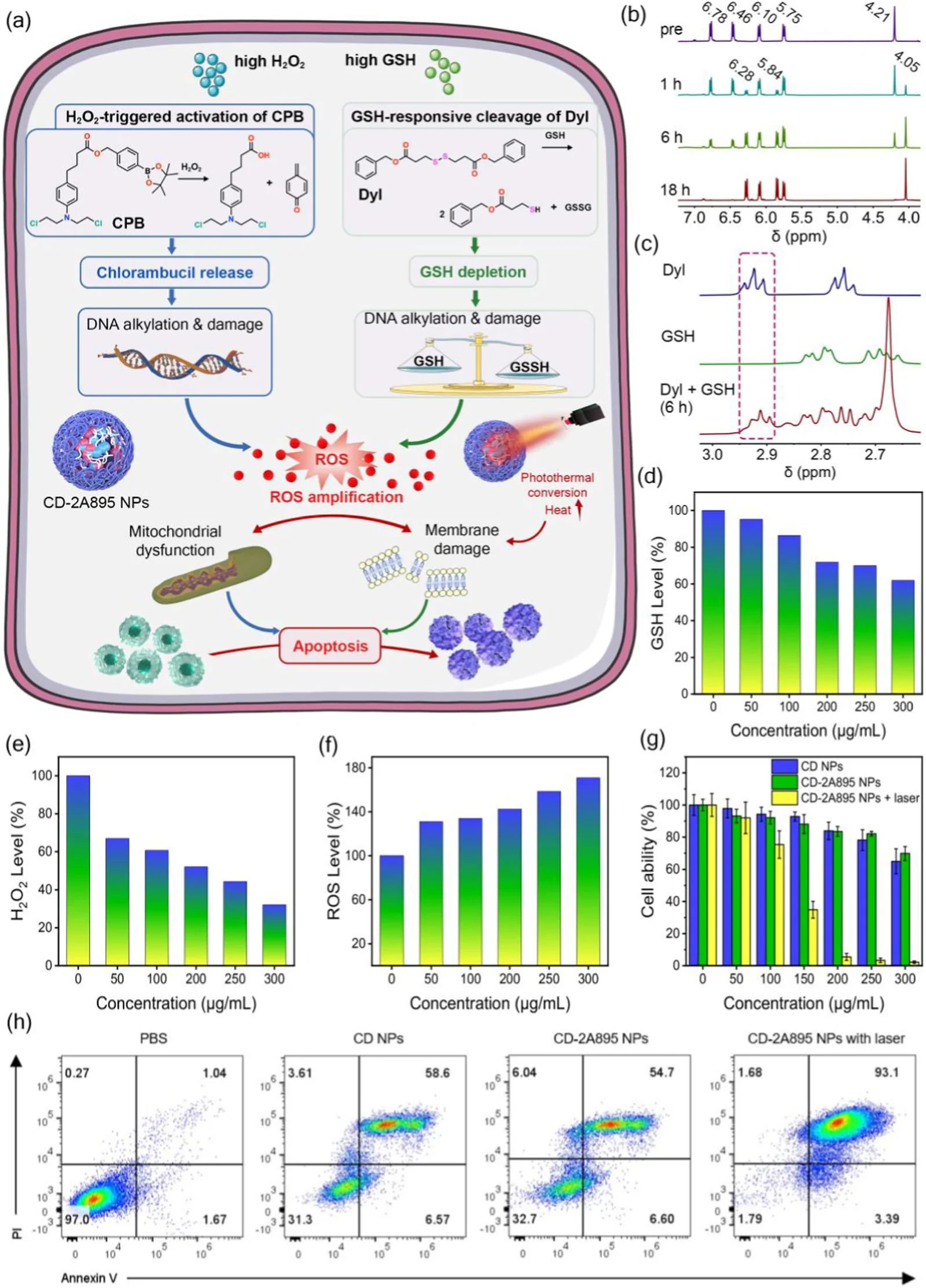
(a) Illustration of H_2_O_2_/GSH dual-responsive therapeutic mechanism of CD-2A895 NPs. (b) Time-dependent ^1^H-NMR spectra of CPB after the addition of H_2_O_2_. (c) ^1^H-NMR spectra of Dyl, GSH, and the reaction mixture of Dyl with GSH. (d-f) Cellular GSH consumption, H_2_O_2_ depletion, and ROS accumulation in 4T1 cells upon nanoparticle treatment. (g) Viability of 4T1 cells treated with CD NPs or CD-2A895 NPs under dark conditions or 808 nm laser irradiation. (h) Distribution of viable and apoptotic 4T1 cells assessed by flow cytometry following the indicated treatments.

CPB responds to H_2_O_2_ through oxidative C-B bond cleavage, generating *p*-quinone methide capable of irreversible GSH scavenging, whereas Dyl consumes GSH via a thiol-disulfide exchange reaction (Fig. 3a) [40,50]. The appearance of characteristic p-quinone methide signals at 4.05, 5.84, and 6.28 ppm after H_2_O_2_ treatment confirms the activation of CPB (Fig. 3b). Likewise, the upfield shifts observed after mixing Dyl with GSH are consistent with thiol–disulfide exchange (Fig. 3c). Together, these NMR results show that both prodrugs preserve their H_2_O_2_- and GSH-responsive activation mechanisms after nanoparticle fabrication.

CD-2A895 NPs exhibited an average hydrodynamic diameter of 169.3 ± 77.0 nm and a surface potential of −17.83 mV (Fig. S37), which is suitable for cellular uptake and tumor accumulation. Their effects on intracellular redox regulation were then evaluated in 4T1 cells. Increasing concentrations of CD-2A895 NPs progressively reduced intracellular GSH and H_2_O_2_ levels while simultaneously increasing ROS production (Fig. 3d–f). These concentration-dependent changes are consistent with activation of the responsive prodrug matrix, where GSH consumption and H_2_O_2_-triggered chlorambucil release jointly shift the intracellular redox balance toward a more oxidative state [43,51].

The biological consequences of this redox modulation were evaluated by MTT assay and flow cytometry. Without laser irradiation, both CD NPs and CD-2A895 NPs produced concentration-dependent cytotoxicity (Fig. 3g), indicating that intracellular prodrug activation alone was sufficient to suppress cell growth. Under 808 nm laser irradiation, CD-2A895 NPs produced a much greater reduction in cell viability across the entire concentration range, consistent with an additional photothermal contribution. Flow cytometry revealed the same trend (Fig. 3h). The apoptotic population increased from 1.04% in the PBS group to 58.6% and 54.7% after treatment with CD NPs and CD-2A895 NPs, respectively, and further increased to 93.1% following laser irradiation of CD-2A895 NPs. These cellular responses are consistent with the cooperative action of redox-responsive chemotherapy and photothermal therapy enabled by the functional matrix.

### 2.4 Multimodal NIR-II imaging

**Fig. 4:**
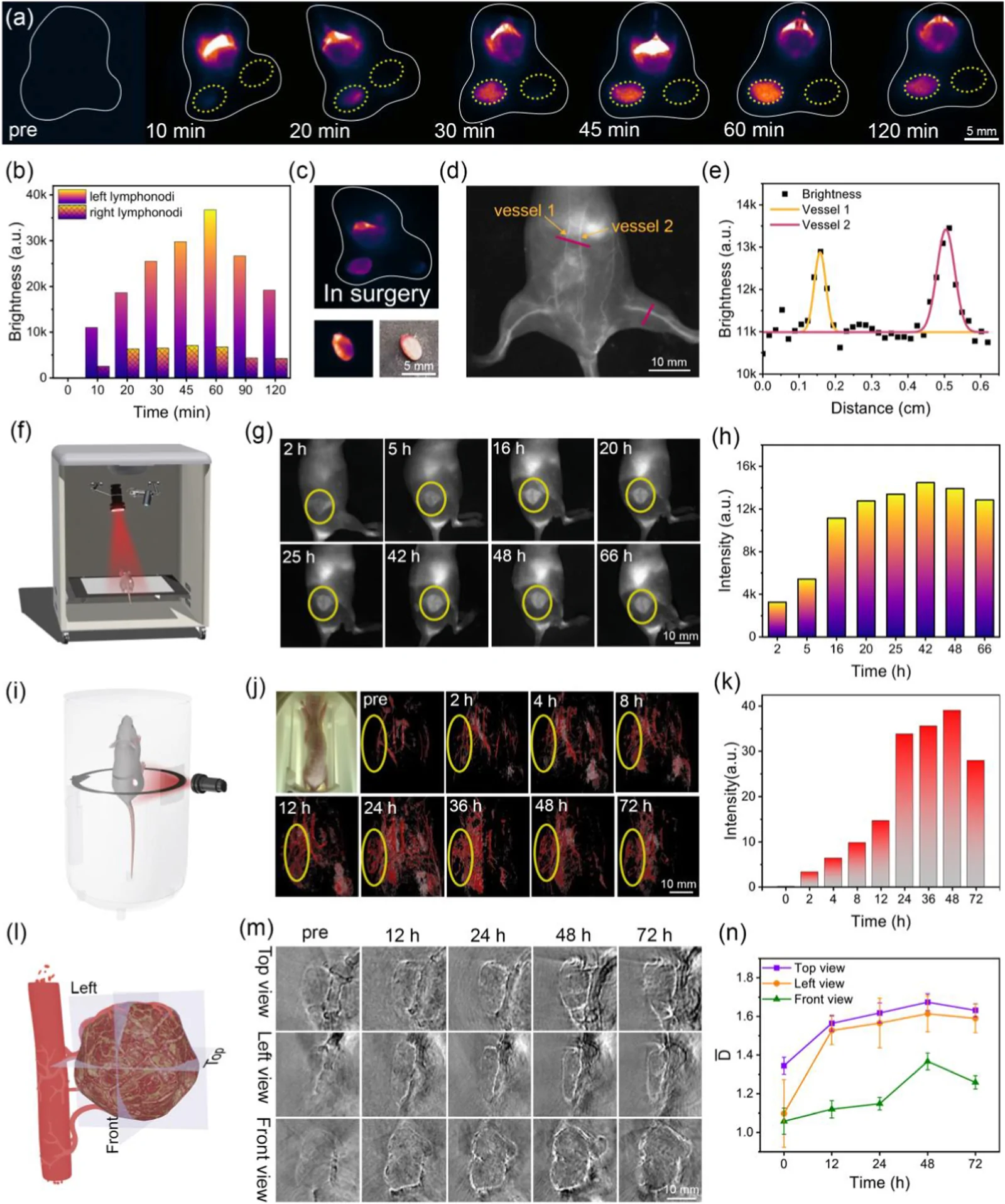
(a) Visualization of superficial cervical lymph nodes under 980 nm excitation (100 mW/cm^2^, 200 ms exposure, LP1000 filter). (b) Fluorescence intensity of superficial cervical lymph nodes extracted from panel (a). (c) Fluorescence-guided surgical resection of the superficial lymph node. (d) Representative NIR-II vascular imaging of abdominal and hindlimb regions (e) Signal cross -sections measured across the vessels indicated in panel (d). (f) Illustration of the experimental setup for NIR-II fluorescence imaging. (g) Sequential imaging of tumor accumulation after nanoparticle administration. (h) Temporal variation of tumor-associated signal intensity. (i) Schematic diagram of the NIR-II photoacoustic (PA) imaging process. (j) Serial PA images acquired at different time intervals following nanoparticle administration. Yellow boxes denote the regions selected for signal analysis. (k) Temporal evolution of PA responses collected from the selected region. (l) Illustration of tumor cross-section selection for PA image analysis. (m) Tumor cross-sections viewed from the top, left, and front directions. (n) Fractal dimension extracted from the corresponding PA images. (For interpretation of the references to colour in this figure legend, the reader is referred to the Web version of this article.)

The in vivo visualization performance of CD-2A895 NPs was first assessed through intralingual injection, followed by optical monitoring in the NIR-II region. The superficial cervical lymph nodes could be clearly identified under 980 nm excitation. Signal intensity increased continuously during the initial hour, reached its highest level at approximately 60 min, and then gradually diminished over the subsequent observation period (Fig. 4a and b), consistent with lymphatic drainage and redistribution of the nanoparticles. In contrast, the contralateral lymph node showed much weaker fluorescence intensity. The clear lymph-node signal further enabled NIR-II fluorescence-guided surgical dissection, demonstrating the potential of CD-2A895 NPs for imaging-guided lymph-node surgical resection (Fig. 4c) [52,53].

Vascular imaging was subsequently conducted using CD-2A895 NPs. Variations in vessel-associated signals provided insight into nanoparticle transport within the circulatory system. Persistent vascular contrast over the observation period suggested extended blood residence, with a circulation half-life estimated to be around 9 h (Fig. S38). High-resolution vascular imaging further revealed clearly distinguishable abdominal and hindlimb blood vessels under an 1100 nm long-pass filter (Fig. 4d). The measured vessel widths were 0.38 mm for hindlimb vessels and 0.16/0.25 mm for abdominal vessels, with corresponding signal-to-background ratios of 1.4, 1.15, and 1.25, respectively (Fig. 4e and S39), confirming the capability of CD-2A895 NPs for NIR-II vascular visualization [4,54].

The biodistribution and tumor-targeting characteristics of CD-2A895 NPs were subsequently assessed in a 4T1 murine tumor model. Tumor-associated NIR-II fluorescence gradually increased after intravenous administration and reached its maximum at approximately 42 h post-injection, indicating efficient tumor accumulation and prolonged retention of CD-2A895 NPs within the tumor microenvironment(Fig. 4f–h). The high tumor-to-background contrast demonstrates the suitability of the nanoparticles as NIR-II fluorescence imaging probes.

Photoacoustic (PA) imaging was subsequently employed to further evaluate their in vivo imaging capability. The PA signal within tumors continuously increased during the first 48 h before gradually declining at 72 h, consistent with the fluorescence imaging results (Fig. 4i–k). Ultrasound-assisted fractal-dimension analysis further revealed an increase in tumor structural complexity following nanoparticle accumulation(Fig. 4l–n), providing quantitative evidence for effective intratumoral localization. Together, these results demonstrate that the enhanced aggregate-state photophysics achieved through functional matrix engineering translate directly into robust multimodal NIR-II fluorescence and photoacoustic imaging performance in vivo [55].

### 2.5 *In vivo* chemo/photothermal therapy

**Fig. 5:**
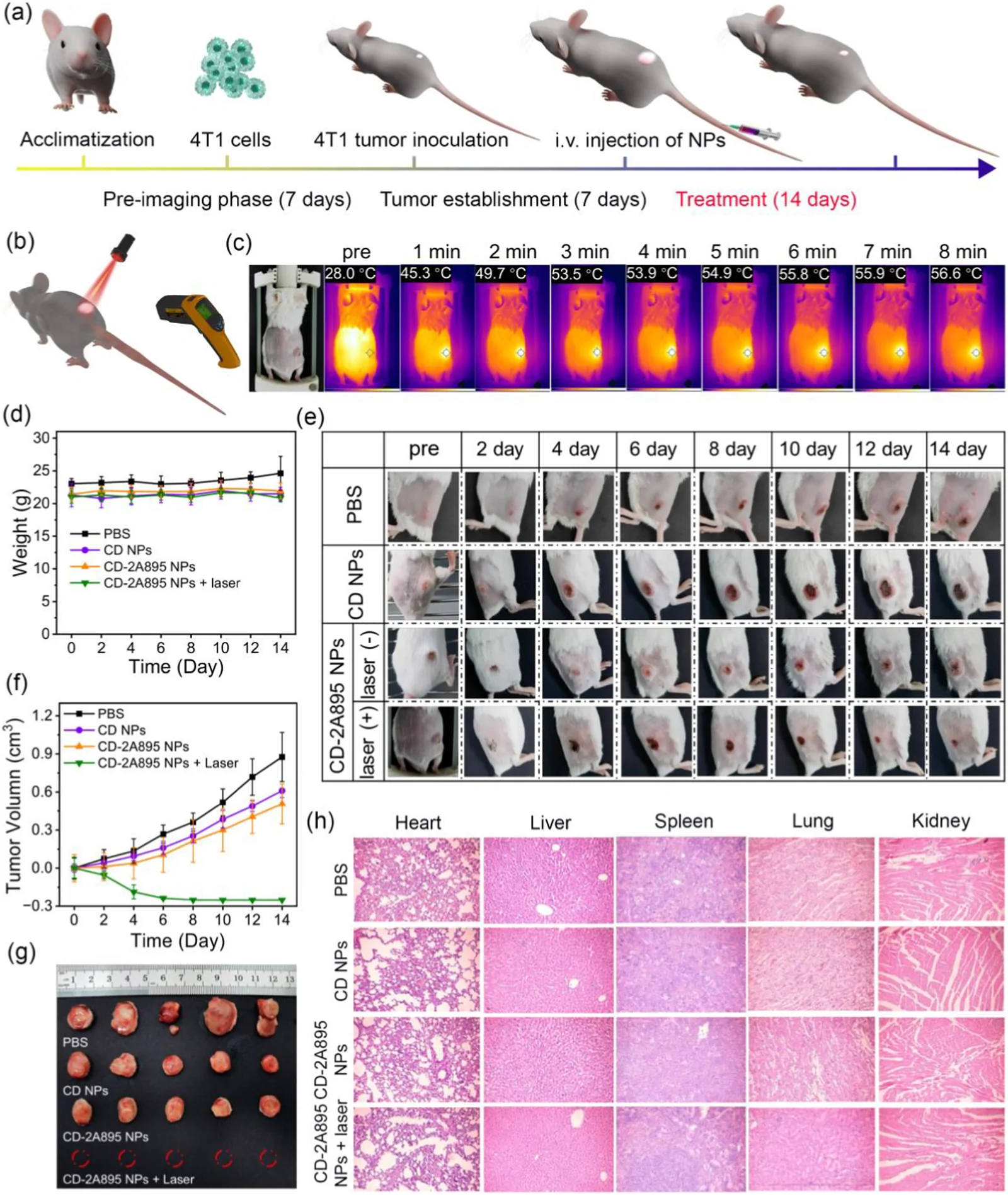
(a) Experimental design for in vivo therapeutic evaluation. (b) Illustration of photothermal treatment and infrared thermal imaging. (c) Infrared thermal images of tumors recorded with a commercial infrared camera. (d) Mouse body weight monitored throughout treatment. (e) Sequential photographs of tumors during therapy. (f) Changes in relative tumor volume for each treatment group. (g) Excised tumors collected at the end of treatment. (h) H&E histological sections of major organs from different treatment groups.

Guided by the imaging results, tumors were irradiated with an 808 nm laser 48 h after nanoparticle administration, corresponding to the time of maximal tumor accumulation. Laser irradiation rapidly increased the tumor temperature in the CD-2A895 NP group, whereas only minimal heating was observed in the control groups (Fig. 5a–c and Fig. S40). Throughout the 14-day treatment period, no appreciable body-weight loss was observed in any group, suggesting good systemic tolerance (Fig. 5d).

Clear differences in therapeutic efficacy emerged during tumor growth monitoring (Fig. 5e–g). Tumors in the PBS group grew continuously, while treatment with the TME-responsive CD NPs slowed tumor progression to a limited extent. By comparison, mice receiving CD-2A895 NPs together with 808 nm laser irradiation showed nearly complete suppression of tumor growth, with most tumors disappearing by the end of treatment. Histological analysis was consistent with these observations. Extensive tumor destruction was evident after the combination treatment, whereas the heart, liver, spleen, lung, and kidney showed no obvious pathological abnormalities (Fig. 5h, S41) [[56], [57], [58]]. These in vivo results support the therapeutic advantage of combining redox-responsive chemotherapy with photothermal therapy through the functional matrix while maintaining good biocompatibility.

## 3 Conclusion

In summary, we present functional matrix engineering as an assembly-based strategy for regulating molecular packing in organic NIR-II phototheranostic nanoparticles. Rather than serving as a passive structural spacer, the tumor-microenvironment-responsive prodrug matrix actively reshapes intermolecular interactions during nanoparticle assembly. The structural reorganization is expected to weaken effective intermolecular electronic coupling between neighboring fluorophores, alleviates aggregation-caused quenching, and improves NIR-II fluorescence without compromising photothermal conversion. Molecular dynamics simulations further connect these photophysical changes to disruption of compact π–π packing. The same responsive matrix that regulates molecular packing also activates chemotherapy through GSH- and H_2_O_2_-responsive prodrugs in the tumor microenvironment. Together with the improved photophysical performance, this dual functionality enables effective multimodal imaging-guided chemo-photothermal therapy while preserving good biosafety. This work illustrates how engineering the matrix, rather than the fluorophore alone, provides a versatile route to control aggregate-state photophysics while integrating therapeutic functions within molecular assemblies.

## CRediT authorship contribution statement

**Lei An:** Writing – review & editing, Writing – original draft, Visualization, Software, Project administration, Investigation, Data curation, Conceptualization. **Changjin Ou:** Writing – review & editing, Supervision, Project administration, Methodology, Conceptualization. **Honghuaijin An:** Visualization, Software, Formal analysis. **Hanjun Sun:** Validation, Methodology, Data curation. **Xiaozhou Mou:** Resources. **Xiaochen Dong:** Writing – review & editing, Supervision, Project administration, Funding acquisition. **Yu Cai:** Writing – review & editing, Supervision, Resources, Project administration, Funding acquisition, Conceptualization.

## Declaration of competing interests

The authors declare that they have no competing interests.

## Figures and Tables

**Figure:**
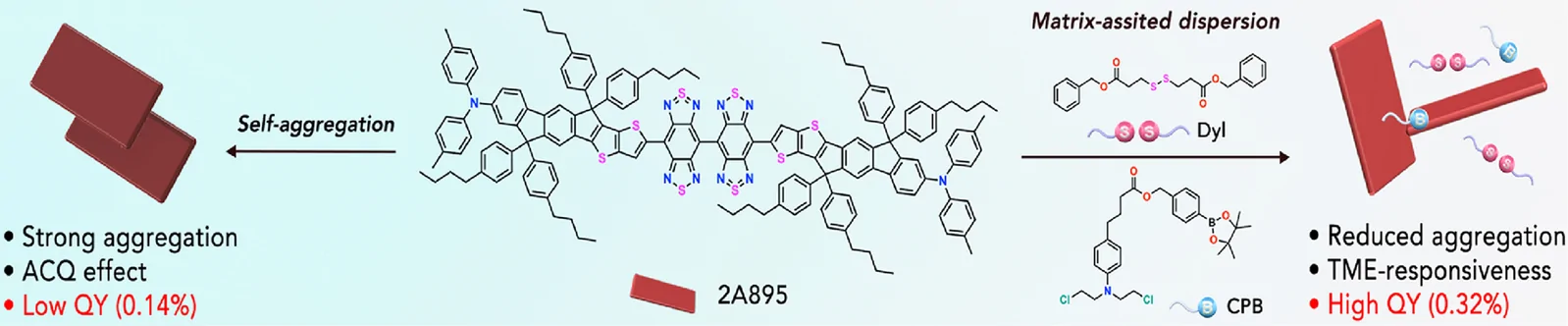


## Data Availability

Data will be made available on request.

## Appendix

Functional matrix engineering transforms self-aggregated NIR-II fluorophores into programmable molecular assemblies. The responsive matrix reorganizes intermolecular organization, suppresses aggregation-caused quenching, enhances NIR-II fluorescence, and simultaneously enables tumor microenvironment-responsive chemo-photothermal therapy.
