# Supplementary material for: Functional matrix engineering enables aggregate-state photophysics in tumor-responsive organic NIR-II phototheranostics
Source: Mater Today Bio. 2026 Aug 19;40:103575. doi: 10.1016/j.mtbio.2026.103575 (PMC13560337; doi:10.1016/j.mtbio.2026.103575)
Supplement: Supplementary file 1 [file mmc1.docx]

Supporting Information

**Functional Matrix Engineering Enables Aggregate-State Photophysics in Tumor-Responsive Organic NIR-II Phototheranostics**

Lei An^1^, Changjin Ou^2^*, Honghuaijin An^1^, Hanjun Sun^3^, Xiaozhou Mou^1^, Xiaochen Dong^3^*, Yu Cai^1^*

Lei An, Honghuaijin An, Xiaozhou Mou, Yu Cai

Center for Rehabilitation Medicine, Rehabilitation & Sports Medicine Research Institute of Zhejiang Province, Department of Rehabilitation Medicine, Cancer Center, Zhejiang Provincial People’s Hospital (Affiliated People’s Hospital), Hangzhou Medical College, Hangzhou, Zhejiang 310014, China

E-mail: iamycai@163.com

Changjin Ou

Institute of Advanced Materials and Flexible Electronics (IAMFE), School of Chemistry and Materials Science, Nanjing University of Information Science and Technology, Nanjing, China

E-mail: 003239@nuist.edu.cn

Hanjun Sun, Xiaochen Dong

State Key Laboratory of Flexible Electronics (LoFE) & Institute of Advanced Materials (IAM), School of Flexible Electronics (Future Technologies), Nanjing Tech University (NanjingTech), Nanjing 211816, China

E-mail: iamxcdong@njtech.edu.cn

**1. Materials and characterization**

All chemicals were purchased from Energy-Chemical Co. Ltd., and the solvents for chemical reactions were used without purification unless otherwise stated. All oxygen and moisture-sensitive reactions were carried out in flame-dried glassware under N_2_ atmosphere. DSPE-PEG2000 was purchased from Bidepharm Co. Ltd. RPMI 1640 Medium cell cultures were purchased from Adamas Co. Ltd. Annexin V-FITC/PI cell apoptosis kit, hydrogen peroxide assay kit (S0038) and reactive sxygen species assay kit based on DCFH-DA (S0033) were purchased from Shanghai Beyotime Biotechnology Co., Ltd. The assay kit for trace reduced glutathione (GSH) was purchased from Nanjing Jiancheng Bioengineering Institute.

^1^H and ^13^C NMR spectra were measured on 400 MHz NMR spectrometers (JEOL 400YH) using CDCl_3_ or DMSO-*d*_6_ as a deuterated solvent and tetramethylsilane (TMS) as an internal standard. ESI-MS spectra were recorded on a Waters G2-S Q-Tof mass spectrometer (Waters Corporation, USA). UV-vis absorption and NIR-II fluorescence spectra were measured by a SHIMADZY UV-3600 plus spectrophotometer and an Edinburgh FLS980 spectrophotometer, respectively. Femtosecond transient absorption spectroscopy was carried out using a Helios ultrafast transient absorption spectrometer. The pump beam was spatially overlapped with the supercontinuum probe and focused into a 2 mm optical cuvette containing the sample solution. The pump–probe delay was scanned over a time range from -5 to 7700 ps. Initial data processing, including chirp correction, was performed with Surface Xplorer. Subsequent kinetic analysis at selected wavelengths was conducted in MATLAB. In this study, classical molecular dynamics (MD) simulations were performed using the Forcite module in Materials Studio 2023 to calculate the radial distribution functions (RDFs) and densities in different systems. The simulation procedure is as follows: First, the initial structure of the electrolyte was constructed using the Amorphous Cell tool in Materials Studio, and geometric optimization was performed using the COMPASS III force field. Subsequently, a 1000 ps equilibrium simulation was performed under the NPT collection, with conditions of 298.15 K, 1 atm, and a time step of 1 fs, and temperature control was achieved using the Nosé thermal bath method. The size and scale were measured via dynamic light scattering (DLS). The cytotoxicity, GSH, H_2_O_2_, and ROS content in cells were determined by Varioskan LUX multifunctional enzyme-linked immunosorbent assay (Thermo Fisher Scientific, Inc.). NIR-II fluorescence imaging used a fluorescence imaging system (Suzhou Near-Optics, China). The infrared imaging adopted the FOCRIC 320Pro series professional handheld thermal imager. NIR-II photoacoustic imaging was performed by the TomoWave LOIS-3D photoacoustic imaging instrument (TomoWave Laboratories, Inc.).

**2. Synthesis of NIR-II dye 2A895**

**Scheme S1.** Synthetic route of NIR-II dye 2A895.

**Synthesis of Compound 1:** Pd(PPh_3_)_4_ (0.185 g, 0.160 mmol, 0.05 equiv.) was added to the deoxygenated THF/Tol solution (20.00 mL/20.00 mL) of 4,4,5,5-tetramethyl-2-(thieno[3,2-b]thiophen-2-yl)-1,3,2-dioxaborolane (C_12_H_15_BO_2_S_2_, 0.850 g, 3.193 mmol, 1 equiv.), diethyl 2,5-dibromoterephthalate (C_12_H_12_Br_2_O_4_, 1.820 g, 4.790 mmol, 1.5 equiv.), and K_2_CO_3_/KF solution (2 M, 3.20 mL, 2 equiv.) under N_2_ atmosphere. The resultant mixture was stirred at 90 °C in the dark for 24 hours. After cooling to R.T., the mixture was extracted three times with dichloromethane (DCM). The organic phase was dried with MgSO_4_ and evaporated in vacuum. The crude product was purified by silica gel column chromatography (PE/EA = 40:1) to afford compound 1 (C_18_H_15_BrO_4_S_2_, 0.630 g, 45 % yield). **^1^H NMR** (400 MHz, Chloroform-*d*) δ 8.01 (s, 1H), 7.91 (s, 1H), 7.40 (d, J = 5.2 Hz, 1H), 7.32 – 7.19 (m, 2H), 4.43 (q, J = 7.2 Hz, 2H), 4.23 (q, J = 7.2 Hz, 2H), 1.41 (t, J = 7.2 Hz, 3H), 1.13 (t, J = 7.2 Hz, 3H). **^13^C NMR** (100 MHz, Chloroform-*d*) δ 166.99, 165.77, 141.81, 140.35, 139.76, 135.64, 135.50, 134.96, 134.00, 133.78, 127.90, 121.34, 119.74, 119.70, 62.23, 62.04, 13.97, 13.59.

**Synthesis of Compound 2:** Pd(PPh_3_)_4_ (0.085 g, 0.074 mmol, 0.05 equiv.) was added to a mixture of compound 1 (C_18_H_15_BrO_4_S_2_, 0.650 g, 1.480 mmol, 1 equiv.), 4-methyl-N-(4-(4,4,5,5-tetramethyl-1,3,2-dioxaborolan-2-yl)phenyl)-N-(p-tolyl)aniline (0.768 g, 1.924 mmol, 1.3 equiv.), K_2_CO_3_/KF solution (2 M, 1.50 mL, 2 equiv.) and tetrahydrofuran/toluene (20.00 mL/20.00 mL) under N_2_ atmosphere. The mixture was stirred at 90 °C in the dark for 24 hours. After cooling to R.T., the mixture was extracted three times with DCM. The organic phase was dried with MgSO_4_ and evaporated in vacuum. The crude materials were purified by silica gel column chromatography (PE/EA = 40:1) to afford compound 2 (C_38_H_33_NO_4_S_2_, 0.700 g, 76 % yield) as yellow solid. **^1^H NMR** (400 MHz, Chloroform-*d*) δ 7.90 (s, 1H), 7.75 (s, 1H), 7.43 – 7.33 (m, 1H), 7.30 – 7.17 (m, 2H), 7.20 – 6.96 (m, 12H), 4.28 – 4.14 (m, 4H), 2.33 (s, 6H), 1.16 – 1.09 (m, 6H). **^13^C NMR** (100 MHz, Chloroform-*d*) δ 168.73, 168.48, 148.54, 145.51, 142.98, 141.88, 140.14, 139.74, 134.52, 133.75, 133.30, 132.83, 132.70, 132.66, 131.84, 130.36, 129.47, 127.46, 125.26, 122.01, 119.76, 119.33, 61.69, 61.46, 20.67, 13.61.

**Synthesis of Compound 3:** n-BuLi (1.6 M in hexane, 5.10 mL, 8.160 mmol, 8 equiv.) was added dropwise to a solution of 1-bromo-4-butylbenzene (C_10_H_13_Br, 1.739 g, 8.160 mmol, 8 equiv.) in anhydrous THF at -70 ℃ under N_2_ atmosphere. After stirring for another 2 h, compound 2 (C_38_H_33_NO_4_S_2_, 0.700 g, 1.108 mmol) was added. Then the reaction was slowly warmed up to R.T. and stirred overnight. After stirring, the mixture was poured into saturated brine and extracted three times with DCM. The combined organic phase was dried with MgSO_4_ and evaporated in vacuum without further purification. BF_3_·Et_2_O (0.40 mL) was added to a solution of the crude product in DCM (30.00 mL). The mixture was stirred at R.T. for 25 min. Then the mixture was poured into sodium hydroxide solution and extracted three times with DCM. The organic phase was dried with MgSO_4_ and evaporated in vacuum. The crude materials were purified by silica gel column chromatography (PE/DCM = 15:1) to afford compound 3 (C_74_H_73_NS_2_, 0.780 g, 70%) as yellow solid. **^1^H NMR** (400 MHz, Chloroform-*d*) δ 7.66 (s, 1H), 7.45 (d, J = 8.4 Hz, 1H), 7.39 (s, 1H), 7.32 – 7.20 (m, 2H), 7.20 – 7.13 (m, 5H), 7.12 – 7.00 (m, 12H), 7.00 – 6.86 (m, 9H), 2.56 (q, J = 7.9 Hz, 8H), 2.29 (s, 6H), 1.61 – 1.51 (m, 8H), 1.35 (dt, J = 14.8, 7.4 Hz, 8H), 0.91 (dtd, J = 8.4, 7.3, 1.0 Hz, 12H). **^13^C NMR** (100 MHz, Chloroform-*d*) δ 153.67, 153.56, 151.48, 147.93, 146.27, 145.71, 143.97, 143.60, 141.98, 141.50, 141.03, 138.70, 137.20, 134.40, 134.19, 132.54, 130.10, 128.76, 128.59, 128.54, 128.46, 126.56, 124.52, 122.56, 121.24, 120.68, 120.60, 117.45, 117.25, 64.65, 62.88, 35.15, 35.11, 33.45, 33.37, 22.34, 22.29, 20.62, 13.81, 13.78.

**Synthesis of 2A895:** Compound 3 (0.709 g, 0.681 mmol, 2 equiv.) was dissolved in 10.00 mL anhydrous THF at -40 ℃ under N_2_ atmosphere, and then added n-BuLi (1.6M in hexane, 0.85 mL, 1.362 mmol, 4 equiv.) dropwise. After stirring for 2 h, 2-isopropoxy-4,4,5,5-tetramethyl-1,3,2-dioxaborolane (C_9_H_19_BO_3_, 0.35 mL, 1.703 mmol, 5 equiv.) was added. This reaction was warmed up to R.T. and stirred overnight. Then, THF was evaporated to obtain the crude product of boronic acid ester. For a Suzuki reaction, 2Br-BBT (C_6_Br_2_N_4_S_2_, 0.120 g, 0.341 mmol, 1 equiv.), Pd(PPh_3_)_4_ (0.040 mg, 0.034 mmol, 0.1 equiv.), K_2_CO_3_/KF solution (2 M, 3.40 mL, 2 equiv.) and the crude product were added into deoxygenated Tol/THF (15.00 mL/15.00 mL) under N_2_ atmosphere. The mixture was stirred at 90 °C in the dark for 24 hours. After cooling to R.T., the mixture was poured into water and extracted three times with DCM. The organic phase was dried with MgSO_4_ and evaporated in vacuum. The crude product was subjected to column chromatography using silica gel to afford compound 2A895 (C_160_H_144_N_10_S_8_, 0.100 g, 12% yield). **^1^H NMR** (400 MHz, Chloroform-*d*) δ 9.50 (s, 2H), 7.70 (s, 2H), 7.57 – 7.45 (m, 4H), 7.35 – 7.20 (m, 14H), 7.13 (d, J = 8.3 Hz, 16H), 7.08 – 6.96 (m, 14H), 6.95 – 6.88 (m, 8H), 2.65 – 2.51 (m, 16H), 2.30 (s, 12H), 1.42 – 1.29 (m, 32H), 0.99 – 0.77 (m, 24H). **^13^C NMR** (100 MHz, Chloroform-*d*) δ 155.37, 154.89, 154.32, 152.23, 151.35, 148.79, 148.69, 147.49, 146.05, 144.67, 143.87, 142.66, 142.02, 141.25, 140.64, 140.19, 137.06, 134.50, 133.14, 130.56, 129.41, 129.01, 125.06, 122.88, 121.45, 121.27, 118.44, 117.98, 113.51, 65.08, 63.46, 35.62, 35.53, 33.87, 33.80, 22.78, 22.72, 21.06, 14.23, 14.20.

3. Synthesis of matrix molecules

**Scheme S2.** Synthetic route for matrix molecules CPB and Dyl.

**Synthesis of CPB:** 4-(4-(bis(2-chloroethyl)amino)phenyl)butanoic acid (CLB) (C_14_H_19_C_l2_NO_2_, 0.200 g, 0.657 mmol, 1 equiv.), 2-(4-(bromomethyl)phenyl)-4,4,5,5-tetramethyl-1,3,2-dioxaborolane (C_13_H_18_BBrO_2_, 0.372 g, 1.253 mmol, 2 equiv.) and K_2_CO_3_ (0.182 g, 1.253 mmol, 2 equiv.) were added into a round flask under air. DMSO (20.00 mL) was added to the reaction mixture, and the reaction was stirred at R.T. for 7 hours. After stirring, the reaction was quenched with saturated brine and subsequently extracted using DCM. The organic phase was dried over MgSO_4_, and following filtration, the solvent was evaporated. The crude product was purified by column chromatography to yield the product CPB (C_27_H_36_BCl_2_NO_4_, 0.232 g, 68%) as a yellow oil. **^1^H NMR** (400 MHz, Chloroform-*d*) δ 7.80 (d, J = 7.9 Hz, 2H), 7.34 (d, J = 7.8 Hz, 2H), 7.04 (d, J = 8.6 Hz, 2H), 6.60 (d, J = 8.6 Hz, 2H), 5.11 (s, 2H), 3.73 – 3.66 (m, 4H), 3.64 – 3.57 (m, 4H), 2.54 (t, J = 7.6 Hz, 2H), 2.37 (t, J = 7.5 Hz, 2H), 1.92 (p, J = 7.5 Hz, 2H), 1.34 (s, 12H). **^13^C NMR** (100 MHz, Chloroform-*d*) δ 173.96, 144.76, 139.51, 135.43, 130.87, 130.11, 127.66, 112.38, 84.01, 66.04, 53.56, 40.42, 33.82, 33.47, 29.56, 24.69.

**Synthesis of Dyl:** 3,3'-disulfanediyldipropionic acid (C_6_H_10_O_4_S_2_, 1.000 g, 4.756 mmol, 1 equiv.) was dispersed in a dry single-neck flask containing 20.00 mL DCM and two drops of DMF under air atmosphere. The solution was cooled to 0 °C, and oxalyl chloride (C_2_Cl_2_O_2_, 2.410 g, 19.024 mmol, 4 equiv.) was carefully dropped in portions. After complete addition, the reaction mixture was allowed to reach R.T. and stirred for 3 hours. Subsequently, the excess oxalyl chloride was removed by vacuum distillation at 45 °C, yielding a crude product that was used directly in the next step without requiring further purification. Using the same flask containing the crude product from previous step, 20.00 mL DCM and K_2_CO_3_ (2.629 g, 19.024 mmol, 4 equiv.) were added. Following this, a DCM solution of phenylmethanol (C_7_H_8_O, 2.057 g, 19.024 mmol, 4 equiv.) was introduced dropwise. The mixture was maintained at R.T. overnight to allow for complete reaction. After reaction, the mixture was concentrated under reduced pressure. The resulting residue was then purified by column chromatography, yielding the product (C_20_H_22_O_4_S_2_, 1.000 g, 54%) as a colorless transparent liquid. **^1^H NMR** (400 MHz, Chloroform-*d*) δ 7.41 – 7.29 (m, 10H), 5.14 (s, 4H), 2.85 (dt, J = 60.8, 7.1, 0.9 Hz, 8H). **^13^C NMR** (100 MHz, Chloroform-*d*) δ 172.12, 136.06, 128.97, 128.73, 128.71, 66.67, 33.96, 32.88.

**4. Solvatochromic effect**

Interactions between solvent and fluorescent molecule that affect energy gap between ground state and excited state. The energy gap is closely related to refractive index and dielectric constant of the solvent. According to the Lippert-Mataga equation, ^[1]^ the maximum emission energy of charge transfer band depends linearly on solvent orientation polarization as shown in the following equation:

$$\tilde{v}_{abs}-\tilde{v}_{em}\approx\frac{2\left( \mu_{E}-\mu_{G} \right)^{2}}{hcr^{3}}\Delta f+Const.$$

Where $\tilde{v}_{abs}$ and $\tilde{v}_{em}$ are wavenumber of absorption and emission peaks; $\mu_{E}$ and $\mu_{G}$ are dipole moments of fluorescent molecules in the ground state and excited state; h, c, and r are the Plank constant, light velocity, and radius of the Onsager cavity, respectively; $\Delta f$ refers to the solvent orientation polarization that could be calculated by following equation:

$$\Delta f=\left( \frac{\varepsilon-1}{2\varepsilon+1}-\frac{n^{2}-1}{2n^{2}+1} \right)$$

Where ε and n are the dielectric constant and refractive index of solvent.

In experiment related to this work, we used solvents (ε, n) such hexane (1.89, 1.388), 1,4-dioxane (2.25, 1.424), toluene (2.38, 1.4969), tetrahydrofuran (7.58, 1.4072), chloroform (4.81, 1.4458), and DCM (8.93, 1.4241). The absorption peak and emission peak of 2A895 in solvents were summarized in Table S1.

**5. fsTA Data Analysis and Kinetic Modeling**

Prior to kinetic analysis, the femtosecond transient absorption (fsTA) data were preprocessed by removing invalid wavelength entries, reordering the time and wavelength axes, and retaining only delay times greater than 0.2 ps in order to minimize the influence of coherent artifacts and the instrument response around time zero (IRF = 120 fs). Singular value decomposition (SVD) was then applied to the truncated dataset, showing that the transient absorption response was dominated by only a few principal components and thus supporting the use of a low-dimensional kinetic model for global analysis. On this basis, 2-step and 3-step sequential models were compared, and the 3-step sequential model was found to provide a markedly better fit, with lower residual sum of squares and root mean square error values, indicating that in addition to two dominant ps-scale relaxation processes, a weak long-lived component was required to account for the late-time residual signal. Sequential reconstruction using fixed lifetimes further resolved three distinguishable kinetic components, of which the first two exhibited much stronger spectral contributions than the third, while the corresponding population evolution showed rapid decay of the first component, rise-and-decay behavior of the second, and gradual accumulation of the third at later times. Combined with time-window averaging and feature-wavelength kinetic analysis, these results demonstrate that signals across different spectral regions can be consistently described by the same set of global lifetimes, indicating that they originate from a common sequential relaxation pathway. Overall, the excited-state dynamics of this NIR-II fluorophore are mainly governed by two consecutive ps-scale relaxation processes, with time constants of approximately 1.7 ps and 5.7 ps, corresponding to the initial stabilization of the excited state followed by further conformational/electronic relaxation, while only a weak ns-scale long-lived tail remains at later times.

**6. Fabrication of matrix-2A895 NPs**

In a general procedure, matrix (CPB and Dyl) and NIR-II molecule 2A895 were dissolved in THF with different concentrations. The matrix and 2A895 solution were placed into centrifuge tubes and DSPE-PEG2000 was added to each tube in a quantity that was 10 times the mass of the initial materials. Subsequently, the mixture was slowly injected in deionized (DI) water. Following this, the THF was evaporated under reduced pressure at 40℃. The resulting nanoparticles were stored at ~4°C for further use. The particle size and zeta potential of NPs were determined using dynamic light scattering (DLS) measurements. The NPs were excited under a 980 nm laser, and their fluorescence images were captured at various time points using NIR fluorescence imaging system. The photo-stability of the NPs was assessed by observing changes in fluorescence brightness over time.

**7. Relative determination of photoluminescence quantum yield (PLQY)**

According to reference dye FT-BBT has a PLQY of 19% in toluene at 808 nm reported by published works,^[2]^ relative PLQY of experimental samples was measured by the following equation:

$$\text{Φ}_{\text{sample}}\text{=}\text{Φ}_{\text{FT-BBT}}\text{×}\frac{\text{Slope}_{\text{sample}}}{\text{Slope}_{\text{FT-BBT}}}\text{×}\frac{\text{n}_{\text{sample}}^{\text{2}}}{\text{n}_{\text{toluene}}^{\text{2}}}$$

where n is the refractive index of the dispersion medium (n_Tol_ = 1.4962, n_DCM_ = 1.4242, n_water_ = 1.333).

In details, firstly, measuring the UV-vis absorption spectrum of five gradient concentrations of samples, and maximum absorbance at 808 nm was recommended to no exceed 0.15. Secondly, the fluorescence images were captured by NIR-II image system after being excited by an 808 nm (12.5 mW/cm^2^, 20 ms, LP900 filter). Then, plotting absorbance at 808 nm against with fluorescence brightness collected by ImageJ and the slopes could be fitted by Origin2023 in a linear function. Lastly, guided by the equation, relative fluorescence quantum yield of experimental samples could be calculated by comparing their slopes with FT-BBT in toluene.

**8. Photothermal conversion efficiency (PCE)**

Photothermal conversion efficiency (PCE) is a parameter that measures the absorption of light energy by materials and conversion into thermal energy. The aqueous solution of CD-2A895 NPs (100 μg/mL, 1.0 mL), 2A895 NPs (50 μg/mL, 1.0 mL), and water (1.0 mL) in quartz dish were irradiated by 808 nm laser with power of 1.0 W/cm^2^ for 10 minutes. Then, turning off the 808 nm excitation and cooling the sample at room temperature for 15 minutes. Temperature change of samples was captured by an IR-thermal camera during experiment. The temperature was recorded every 15 seconds.

The PCE was calculated by following equation:

$$\eta=\frac{hs\left( T_{Max}-T_{Surr} \right)-Q_{Dis}}{I(1-{10}^{{-A}_{\lambda}})}$$

Where h is the heat-transfer co-efficient; s is the container’s surface area; Q_Dis_ is the heat dissipation due to the light absorption of container and water; A_λ_ is the absorbance of materials at 808 nm (λ = 808 nm); and I is the laser power density (1 W/cm^2^). The hs can be calculated by cooling curve:

$$hs=\frac{\sum_{i} m_{i}C_{pi}}{\tau_{s}}$$

where the value of m in this work is 1 g; C is the heat capacity of water; τ_s_ is time constant of sample system. The τ_s_ can be calculated by the following equation:

$$\tau_{s}=-\frac{t}{\ln\theta}$$

Where t is time (unit: second); θ is driving force of temperature. The θ can be calculated by following equation:

$$\theta=\frac{T-T_{Surr}}{T_{Max}-T_{Surr}}$$

Where T is testing temperature; T_Surr_ is environmental temperature; T_Max_ is maximum testing temperature.

In addition, Q_Dis_ is energy of water absorbed that can be calculated by the following equation:

$$Q_{Dis}=\frac{cm\Delta T}{t}$$

Where c is heating capacity of water; m is mass of water (1 g); ∆T is maximum value of temperature change after absorbing laser energy. In this work, the temperature of water increased by 3.2 °C within 600 s of continuous excitation by 808 nm laser (1.0 W/cm^2^). Therefore, the Q_Dis_ is 0.00224. The data mentioned above is organized into Table S2.

In addition, during the temperature depended on power density experiment, the CD-2A895 NPs of 100 μg/mL were excited by 808 nm laser at different power densities (0.4, 0.6, 0.8, 1.0, and 1.2 W/cm^2^) for 10 min. And for the temperature depended on concentration experiment, the CD-2A895 NPs were irradiated by 808 nm laser at different concentrations (20, 40, 60, 80, and 100 μg/mL). The temperature of sample was recorded every 30 seconds.

**9. Determination of GSH, H_2_O_2_, ROS content in cells**

4T1 cells were cultured on 96-well cell plates using RPMI 1640 medium. The experiment included a control group and three treatment groups (CD NPs, CD-2A895 NPs, CD-2A895 NPs + laser), with different concentration of 0, 20, 40, 60, 80, and 100 μg/mL. All group were incubated at 37°C and 5% CO_2_ atmosphere. After incubation for 12 hours, tests were conducted to measure GSH and H_2_O_2_ content. Additionally, ROS testing was performed after 6 hours of incubation. All three tests were performed using a multifunctional microplate reader, adhering strictly to the manufacturer’s protocols.

**10. MTT assay and cytotoxicity assays**

4T1 cells were cultured in a 96-well plate and exposed to varying concentrations of CD NPs and CD-2A895 NPs (0, 50, 100, 150, 200, 250, and 300 μg/mL). The control group was cultivated without any nanomaterials. Both the control group and experimental groups were maintained in the dark and 37°C with 5% CO_2_. The group exposed to laser underwent unique irradiation (808 nm, 0.6 W/cm^2^) for 5 min. Following this, MTT in PBS (5.0 mg/mL, 20 μL) was introduced to each well and allowed to incubate for a 4 h reaction. Subsequently, the culture medium was removed, and 200 μL DMSO was added into each well. Finally, the absorbance was measured at 492 nm using a multifunctional microplate reader.

**11. Cell apoptosis experiment**

4T1 cells were cultured on 6-well cell plate with RPMI 1640 medium. Control group and three treatment groups (CD NPs, CD-2A895 NPs, CD-2A895 NPs + laser, concentration: 200 μg/mL) were incubated at 37°C, 5% CO_2_ atmosphere for 24 h. Prior to staining, the CD-2A895 NPs + laser group was irradiated by 808 nm laser at 0.6 W/cm^2^ for 7 minutes. Subsequently, all four groups were stained with both Annexin V-FITC and PI, following the manufacturer’s protocols. Flow cytometry was performed after digesting the 4T1 cells from the 6-well plate.

**12. *In vivo* NIR-II fluorescence and photoacoustic imaging**

The female BALB/c mice (14-18 g weight, five weeks aged) were purchased from Comparative Medicine Centre of Yangzhou University. All animal experiments were approved and guided by the Zhejiang Provincial People’s Hospital, Affiliated People’s Hospital, Hangzhou Medical College, in compliance with the relevant laws and institutional guidelines. 4T1 cells were inoculated into the right hind leg of the mice with 50 μL of PBS containing 4×106 cells. When the tumor volumes approached 100-150 mm^3^, the following NIR-II fluorescence imaging and photoacoustic imaging experiments.

Using tweezers to gently grasp the mouse's tongue and inject CD-2A895 NPs (1.0 mg/mL, 10 μL) on the lingual side. Then, the fluorescence imaging was performed under 980 nm laser irradiation (100.0 mW/cm^2^, 200 ms, LP1000 filter) to observe the fluorescence of the superficial cervical lymph nodes at various time points (0, 10, 20, 30, 45, 60, and 120 minutes).

For *in vivo* fluorescence imaging of blood vessels, CD-2A895 NPs (1.0 mg/mL, 200 μL) was injected into mice via tail vein, and the fluorescence imaging was performed after the injection under 980 nm laser irradiation (100.0 mW/cm^2^; 200 ms; LP1000 filter). The full width at half maximum (FWHM) of fluorescence intensity of vessels was fitted using a Gaussian function to calculate the vascular width. The pharmacokinetic testing also used the same method and parameters.

For *in vivo* fluorescence imaging of tumor, CD-2A895 NPs (1.0 mg/mL, 200 μL) was intravenously injected into mice, and the fluorescence imaging was performed under 980 nm laser irradiation (100.0 mW/cm^2^, 200 ms, LP1000 filter) at different time intervals.

An aqueous solution of CD-2A895 NPs (1.0 mg/mL, 200 μL) was intravenously injected into BALB/c-nu mice inoculated with 4T1 tumor, in vivo photoacoustic imaging was carried out on TomoWave LOIS-3D photoacoustic imaging system at various time intervals (0, 2, 4, 8, 12, 24, 36, 48, and 72 h). During the photoacoustic imaging process, nude mice was fixed onto a scaffold and continuously administered gas anesthesia to ensure a stable imaging environment. The mice were placed in an imaging chamber filled with water and rotated according to a predetermined program, with a step size of 480 and a scanning frequency of 16. The signals excited by a pulsed laser (895 nm) were received by a circular detector. After scanning, the photoacoustic data were analyzed using LOIS View 3D and ImageJ.

The fractal dimension was utilized to assess the shape integrity of the tumors from three different views. A larger fractal dimension indicates a more complex shape with greater detail. We employed the FracLac plugin of ImageJ software to measure the fractal dimension using binary images.

**13. *In vivo* chemo-photothermal therapy**

In this study, once the tumor volume reached approximately 100 cm^3^, the mice were randomly divided into four groups. The control group received PBS injection (200 μL). The other three treatment groups received injections as follows: CD NPs (1.0 mg/mL, 200 μL), CD-2A895 NPs (1.0 mg/mL, 200 μL), and CD-2A895 NPs (1.0 mg/mL, 200 μL) with 808 nm laser. After 48 hours of administration, only CD-2A895 NPs + laser group were subjected to an 8 minutes laser irradiation. Record the weight and tumor volume of mice on time every day for 14 days. After 14 days, the mice were euthanized according to ethical requirements, and H&E staining was performed on the main organs and tumors of each group of mice.

**Figure S1.** ^1^H NMR spectrum of compound 1 (400 MHz, CDCl_3_).

**Figure S2.** ^13^C NMR spectrum of compound 1 (100 MHz, CDCl_3_).

**Figure S3**. ^1^H NMR spectrum of compound 2 (400 MHz, CDCl_3_).

**Figure S4.** ^13^C NMR spectrum of compound 2 (100 MHz, CDCl_3_).

**Figure S5.** ^1^H NMR spectrum of compound 3 (400 MHz, CDCl_3_).

**Figure S6.** ^13^C NMR spectrum of compound 3 (100 MHz, CDCl_3_).

**Figure S7.** ^1^H NMR spectrum of 2A895 (400 MHz, CDCl_3_).

**Figure S8.** ^13^C NMR spectrum of 2A895 (100 MHz, CDCl_3_).

**Figure S9.** Mass spectra of 2A985. (a) Chemical formula and molecular weight obtained by Chemdraw20.0. (b) Mass spectra of 2A895.

**Figure S10.** Evaluation of solvatochromic effect of 2A895. (a) Absorption spectra. (b) Emission spectra.

**Figure S11.** Calculation of molar extinction coefficient of 2A895 in DCM at different wavelengths. (a) Absorption spectra. The plot includes absorbance spectra at three different wavelengths: (b) 808 nm, (c) absorption peak 895 nm, and (d) 980 nm.

**Figure S12.** Singular value decomposition (SVD) results of the transient absorption data. (a) Singular values as a function of component number; (b) cumulative energy fraction as a function of component number.

**Figure S13.** (a) Population evolution of the three states obtained from the sequential model. (b–f) Representative kinetic traces at selected probe wavelengths (956, 1035, 1122, 1208, and 1296 nm), shown together with the corresponding global fits.

**Figure S14.** ^1^H NMR spectrum of compound CPB (400 MHz, CDCl_3_).

**Figure S15.** ^13^C NMR spectrum of compound CPB (100 MHz, CDCl_3_).

**Figure S16.** (a) Chemical structure and exact mass of CPB. (b) ESI-MS of CPB. The protonated molecule [M+H]^+^ was observed.

**Figure S17.** ^1^H NMR spectrum of compound Dyl (400 MHz, CDCl_3_).

**Figure S18.** ^13^C NMR spectrum of compound Dyl (100 MHz, CDCl_3_).

**Figure S19.** (a) Chemical structure and exact mass of Dyl. (b) ESI-MS of Dyl. The protonated ion [M+H]^+^ and the sodiated ion [M+Na]^+^ were observed.

**Figure S20.** Fluorescence quantum yield of FT-BBT in toluene is 19%. (a) Absorption spectra of FT-BBT in toluene with different concentrations. (b) Fluorescence image of FT-BBT excited by 808 nm laser with a 900 nm long pass (12.5 mW/cm^2^, 20 ms). (c) Plot of the brightness spectra at five different concentrations.

**Figure S21.** Calculation of relative fluorescence quantum yield of 2A895 in DCM. (a) Absorption spectra of 2A895 in DCM with different concentrations. (b) Fluorescence images of 2A895 in DCM excited by 808 nm laser with a 900 nm long pass (12.5 mW/cm^2^, 20 ms). (c) Plot of the brightness spectra at five different concentrations.

**Figure S22.** Calculation of relative fluorescence quantum yield of 2A895 NPs. (a) Absorption spectra of 2A895 NPs in DI water with different concentrations. (b) Fluorescence images of 2A895 NPs excited by 808 nm laser with a 900 nm long pass (12.5 mW/cm^2^, 20 ms). (c) Plot of the brightness spectra at five different concentrations.

**Figure S23.** Calculation of molar extinction coefficient of 2A895 NPs at different wavelengths. Plot of the absorbance spectra at three different wavelengths: (a) 808 nm, (b) absorption peak 895 nm, and (c) 980 nm.

**Figure S24.** Calculation of relative fluorescence quantum yield of CPB-2A895 50wt% NPs. (a) Absorption spectra of CPB-2A895 50wt% NPs in DI water with different concentrations. (b) Fluorescence images of CPB-2A895 50wt% NPs excited by 808 nm laser with a 900 nm long pass (12.5 mW/cm^2^, 20 ms). (c) Plot of the brightness spectra at five different concentrations.

**Figure S25.** Calculation of molar extinction coefficient of CPB-2A895 NPs at different wavelengths. Plot of the absorbance spectra at three different wavelengths: (a) 808 nm, (b) absorption peak 907 nm, (c) 980 nm.

**Figure S26.** Calculation of relative fluorescence quantum yield of Dyl-2A895 50wt% NPs. (a) Absorption spectra of Dyl-2A895 30wt% NPs in DI water with different concentrations. (b) Fluorescence images of Dyl-2A895 30wt% NPs excited by 808 nm laser with a 900 nm long pass (12.5 mW/cm^2^, 20 ms). (c) Plot of the brightness spectra at five different concentrations.

**Figure S27.** Calculation of molar extinction coefficient of Dyl-2A895 NPs at different wavelengths. Plot of the absorbance spectra at three different wavelengths: (a) 808 nm, (b) absorption peak 933 nm, and (c) 980 nm.

**Figure S28.** Calculation of relative fluorescence quantum yield of CD-2A895 NPs. (a) Absorption spectra of CD-2A895 NPs in DI water with different concentrations. (b) Fluorescence images of CD-2A895 NPs excited by 808 nm laser with a 900 nm long pass (12.5 mW/cm^2^, 20 ms). (c) Plot of the brightness spectra at five different concentrations.

**Figure S29.** Calculation of molar extinction coefficient of CD-2A895 NPs at different wavelengths. Plot of the absorbance spectra at three different wavelengths: (a) 808 nm, (b) absorption peak 903 nm, and (c) 980 nm.

**Figure S30.** (a) UV–vis–NIR absorption spectra of CPB-2A895 NPs with doping contents ranging from 10 to 100 wt%. (b) Dependence of absorbance on doping content and the corresponding linear fitting. (c) Photoluminescence spectra of CPB-2A895 NPs under 980 nm excitation. (d,e) NIR-II fluorescence images of CPB-2A895 NPs with different doping contents under 808 and 980 nm excitation, respectively. (f) Corresponding fluorescence brightness under 808 and 980 nm excitation, recorded using a 1000 nm long-pass filter.

**Figure S31.** (a) UV–vis–NIR absorption spectra of Dyl-2A895 NPs with doping contents ranging from 10 to 100 wt%. (b) Dependence of absorbance on doping content and the corresponding linear fitting. (c) Photoluminescence spectra of Dyl -2A895 NPs under 980 nm excitation. (d,e) NIR-II fluorescence images of Dyl -2A895 NPs with different doping contents under 808 and 980 nm excitation, respectively. (f) Corresponding fluorescence brightness under 808 and 980 nm excitation, recorded using a 1000 nm long-pass filter.

**Figure S32.** Calculation of photothermal conversion efficiency of CD-2A895 NPs and 2A895 NPs. (a) UV-vis absorption spectra. (b) Cooling curve.

**Figure S33.** Concentration dependence and power dependence of photothermal conversion. (a) Plot ΔT versus time at different laser power densities. (b) Plot ΔT versus time at different CD-2A895 NPs concentrations.

**Figure S34.** (a) Photoacoustic (PA) images of CD-2A895 NPs at different concentrations. (b) Linear relationship between PA intensity and nanoparticle concentration.

**Figure S35.** Three-dimensional trajectories of the centroids of four 2A895 molecules (mol1–mol4) during Forcite MD simulations. (a) 2A895 NPs. (b) CD-2A895 NPs. For 2A895 NPs, mol1–mol2: 15.85 ± 0.59 Å and 9.15 ± 6.27°; for CD-2A895 NPs., mol1–mol4: 16.93 ± 1.32 Å and 68.15 ± 15.66°.

**Figure S36.** Optimized dimer configurations and intermolecular interaction energies of (a) CPB and (b) Dyl calculated by density functional theory (DFT).

**Figure S37.** Dynamic light scattering (DLS) analysis of CD-2A895 NPs showing an average hydrodynamic diameter of 169.3 ± 77.0 nm and a zeta potential of −17.83 mV.

**Figure S38.** Fluorescence intensity used for pharmacokinetic calculations. (a) Bright field photo of nude mice and NIR-II fluorescence images of time series excited by 980 nm (0.1 W/cm^2^, 200 ms, and LP1000 filter). (b) Plot of the brightness spectra at different time.

**Figure S39.** Fluorescence intensity for measuring the width of hind limb blood vessels in nude mice. (a) Bright field photo of mice. (b) Plot of brightness spectra at distance.

**Figure S40.** Infrared photothermal images of mice with 4T1 tumors acquired after injection with PBS under 808 nm laser irradiation (0.8 W/cm^2^, 8 min).

**Figure S41.** Tum or H&E staining images of different mouse groups.

**Table S1.** Spectroscopic data of 2A895 in different solvents.

|  | Hexane | Toluene | Dioxane | TCM | THF | DCM |
| --- | --- | --- | --- | --- | --- | --- |
| λ_abs_/nm | 927 | 985 | 868 | 911 | 878 | 895 |
| λ_em_/nm | 1046 | 1046 | 1046 | 1115 | 1128 | 1183 |
| Δ*f* | -0.00473 | 0.01324 | 0.02398 | 0.14829 | 0.20957 | 0.21714 |
| Δ*v*/cm^-1^ | 1227.3 | 1613.0 | 1960.5 | 2008.3 | 2524.3 | 2720.1 |

**Table S2.** Photophysical parameters of 2A895 and NPs

| Sample | ε (10^4^ L·mol^-1^cm^-1^)^a^ | | | PLQY^b^  (%) | ε×PLQY^c^  (L·mol^-1^cm^-1^) |
| --- | --- | --- | --- | --- | --- |
|  | λ_808_ | λ_Max_ | λ_980_ |  |  |
| 2A895 | 2.91 | 4.25 | 2.34 | 0.36 | 153.0/84.1 |
| 2A895 NPs | 2.08 | 2.81 | 1.76 | 0.14 | 39.3/24.6 |
| CPB-2A895 50wt% NPs | 1.57 | 2.38 | 1.52 | 0.30 | 71.4/45.6 |
| Dyl-2A895 30wt% NPs | 1.05 | 1.47 | 1.28 | 0.31 | 45.6/39.7 |
| CD-2A895 NPs | 2.10 | 3.01 | 1.80 | 0.32 | 96.3/57.6 |

**Table S3.** Data of PCE of CD-2A895 NPs (1 mg/mL) and 2A895 NPs (0.5 mg/mL).

| Sample | $\boldsymbol{\tau}_{\boldsymbol{s}}$ | hs | T_Max_-T_Surr_ | Q_Dis_ | A_808_ | $\boldsymbol{\eta}$ |
| --- | --- | --- | --- | --- | --- | --- |
| CD-2A895 NPs | 465.99 | 0.009013 | 21.5 | 0.0224 | 0.372 | 29.8% |
| 2A895 NPs | 392.57 | 0.010699 | 18.5 | 0.0224 | 0.353 | 31.5% |

**References**

[1] Y. Li, Y. Tang, W. Hu, Z. Wang, X. Li, X. Lu, S. Chen, W. Huang, Q. Fan, Adv. Sci. 2023, 10, e2204695.
[2] a) C. Ou, L. An, Z. Zhao, F. Gao, L. Zheng, C. Xu, K. Zhang, J. Shao, L. Xie, X. Dong, Aggregate 2023, 4, e290; b) X. Zhou, K. Zhang, C. Yang, Y. Pei, L. Zhao, X. Kang, Z. Li, F. Li, Y. Qin, L. Wu, Adv. Funct. Mater. 2022, 32.
